# Nasopalatine duct cyst as a delayed consequence of dental implant placement in the anterior maxilla: A case report

**DOI:** 10.34172/japid.2023.022

**Published:** 2023-11-20

**Authors:** Mohammadreza Talebi Ardakani, Behzad Houshmand, Aida Kheiri

**Affiliations:** Department of Periodontics, School of Dentistry, Shahid Beheshti University of Medical Sciences, Tehran, Iran

**Keywords:** Case report, Dental implant, Oral pathology, Xenograft

## Abstract

Dental implants are now the best treatment method to replace missing teeth. However, complications may necessitate further therapeutic interventions because of anatomic limitations and mistakes during surgical procedures. In this case report, a nasopalatine duct cyst (NPDC) due to implant placement was studied. After clinical and radiographic evaluation, unilocular radiolucency with disturbance to the nasopalatine canal was observed. Following that, flap elevation was performed. Subsequently, the cyst was enucleated, and the bone defect was filled with xenograft and further covered with a resorbable membrane. Histopathology results confirmed NPDC as the definite diagnosis. After six months, the defect was completely resolved.

## Introduction

 Dental implants are nowadays considered the most acceptable treatment for replacing missing teeth.^1-3^ Placement of dental implants in the anterior maxilla may sometimes have some limitations from the depth and angulation aspects due to anatomical landmarks, esthetic considerations, and possible bone loss following tooth extraction.^4,5^ The nasal cavity, maxillary sinus, and nasopalatine duct are among landmarks of great importance and may even put implant surgical preparation at risk.^4^

 Nasopalatine duct cyst (NPDC) is the most common non-odontogenic cyst of the oral cavity, with a prevalence of about 1% in the general population and a male predilection with a 3:1 ratio.^6,7^ It is believed that NPDC develops from the proliferation of epithelium remnants of the nasopalatine duct. Moreover, local trauma and infection may provoke these cell remnants to proliferate.^7,8^ Though NPDC is usually asymptomatic, painful swelling and drainage may occur.^8^ This case report presents the diagnosis of NPDC as a result of nasopalatine duct disturbance during implant placement. Also, the surgical approach and histopathological evaluation are further presented.

## Case Report

**Figure 1:**
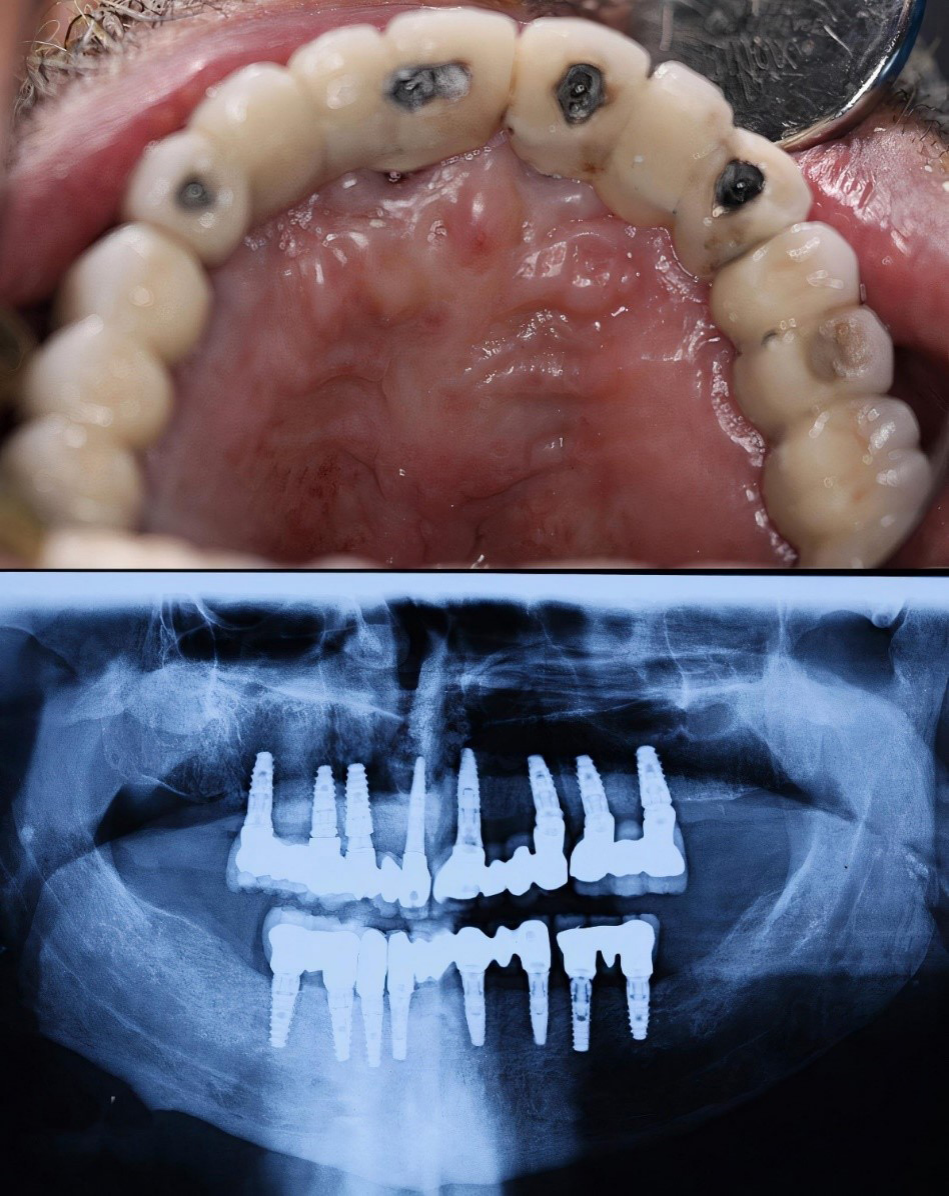
Clinical image and panoramic view of the swelling in the anterior maxilla

 A 68-year-old man was referred to the clinic with a chief complaint of swelling in the anterior palatal portion of the maxilla for the last two months. The patient had no history of systemic problems or medication use. He also mentioned that five years ago, he had undergone full-mouth reconstruction therapy. Intraoral examination revealed a swelling in the midline of the anterior maxilla, near the area where central implants had been inserted (Figure 1). The patient reported no signs of suppuration or pain.

###  Investigation and treatment

**Figure 2:**
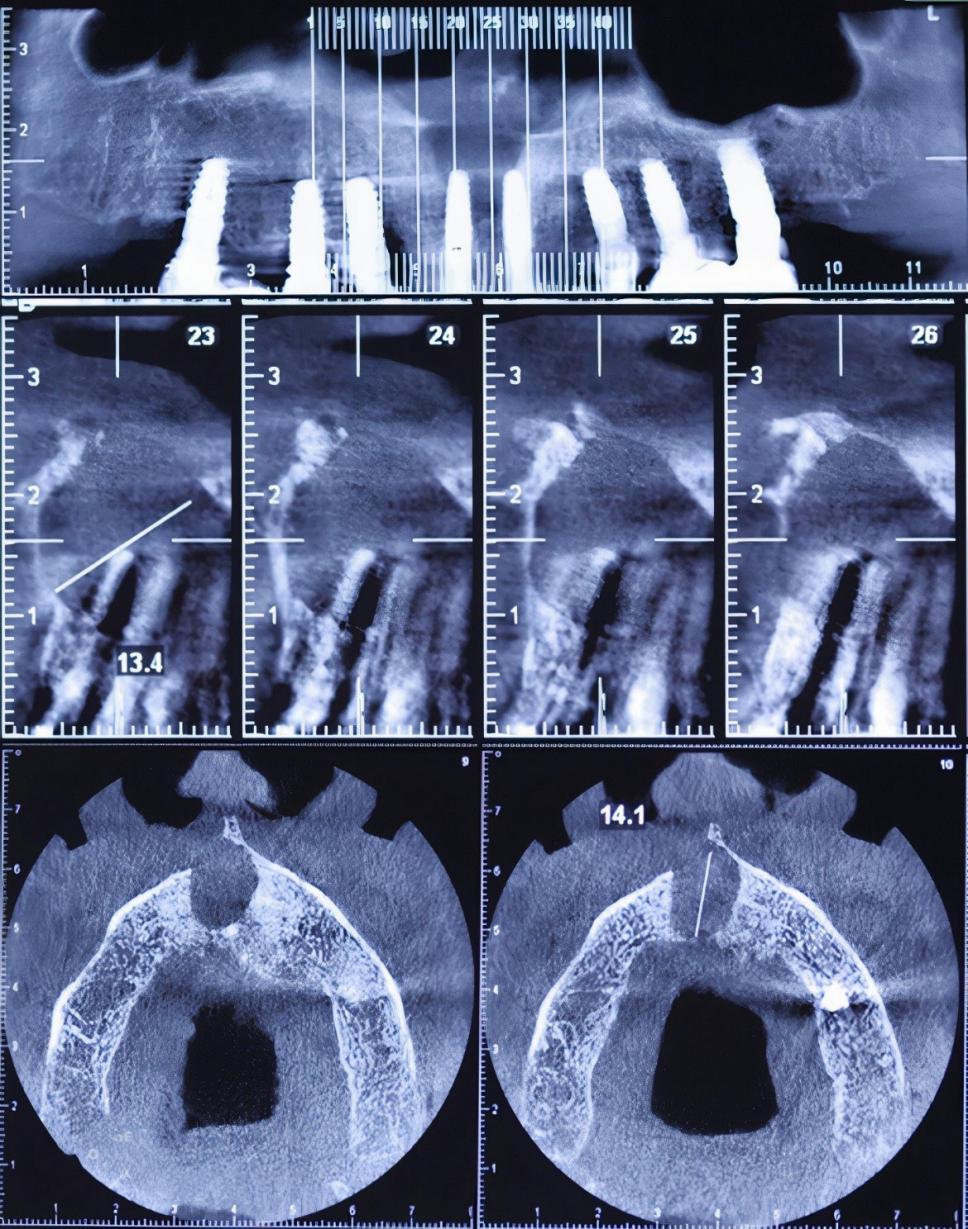
Cone-beam computed tomography (CBCT) imaging of the maxilla. Sections #23 and #24 show the disturbance of the nasopalatine canal and the nasal floor

 A full radiographic examination was requested. Following radiographic evaluation of panoramic radiograph and sections of cone-beam computed tomography (CBCT), an oval-shaped, unilocular radiolucency with a well-defined boundary was observed that not only surrounded the apex of the right central implant but also disturbed the nasopalatine canal and nasal floor (Figures 1 and 2).

 Before undertaking treatment, a detailed description of the treatment procedure was explained to the patient, and informed consent was obtained for the treatment and its publication in an article. The CARE checklist was completely followed in this study.

**Figure 3:**
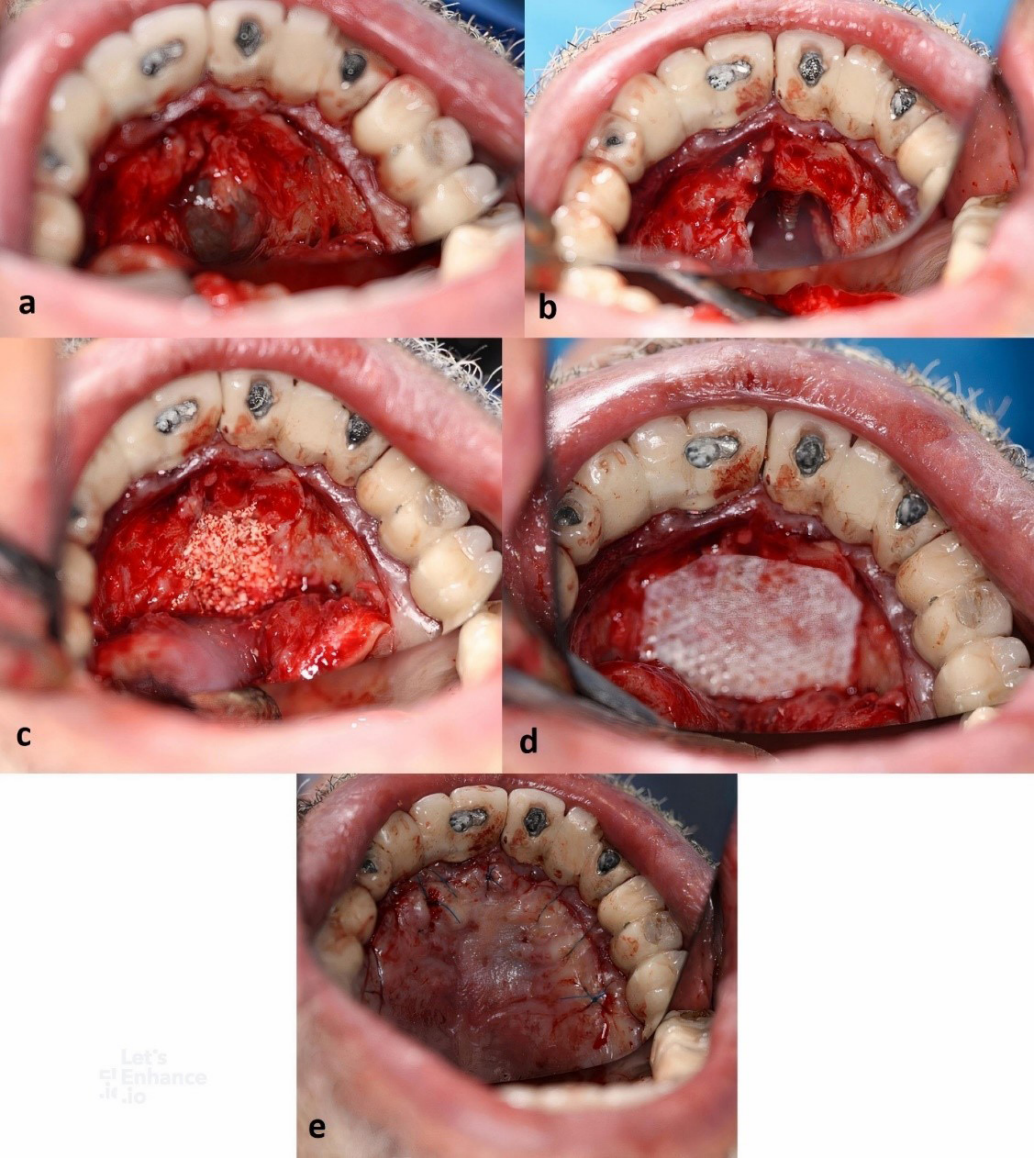
Surgical procedure. A: Flap elevation following submarginal incisions. B: Complete enucleation of the cyst. C: Filling the defect with deproteinized bovine mineral (Bio-Oss, Geistlich pharma^®^). D: Placement of a membrane over the grafted area. E: Primary closure with sutures

 Following local anesthesia (2% lidocaine, epinephrine: 1:100 000), a submarginal incision was performed extending from the right first molar to the left first molar, and a mucoperiosteal flap was elevated. Care was taken to design the flap far enough from the defect. The flap was elevated sufficiently to provide visibility and access to the surgical area (Figure 3A). After detaching the cyst’s walls from the palatal bone, the cyst was completely enucleated (Figure 3B). Since the implant showed no mobility, inflammation, or bone loss in the marginal area, it was left intact. Both bone defect and implant surface were decontaminated with H_2_O_2_ for five minutes. The sample was sent to the Oral and Maxillofacial Pathology Department of Shahid Beheshti Dental School (SBMU) for histopathological assessment.

 The defect was filled with deproteinized bovine mineral (Bio-Oss, Geistlich Pharma^®^) with a particle size of 0.25‒1 mm (Figure 3C). A resorbable membrane fully covered the defect (Figure 3D). The flap was repositioned at its original place and sutured with 5/0 nylon (Dafilon^®^) (Figure 3E). The patient was advised to rinse with 0.12% CHX twice a day for two weeks and take 500-mg amoxicillin capsules three times a day for seven days.

###  Histopathological findings

**Figure 4:**
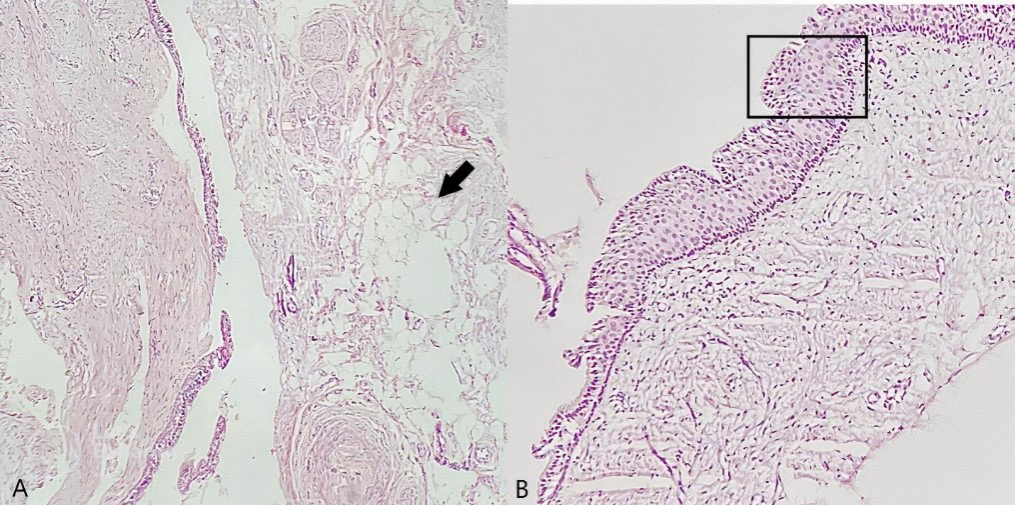
Histologic images of the sample ( × 40). A: The black arrow shows adipose tissue. B: The black rectangle shows the lining cells of the cyst

 Histological evaluations revealed a cystic lesion lined with variable cuboidal, stratified squamous epithelium and focally ciliated columnar epithelium. The cyst wall consisted of fibrovascular tissue, inflammatory cell infiltration, hemorrhage, sections of adipose tissue, and normal bone trabeculae (Figure 4).

###  Final diagnosis

 A nasopalatine duct cyst was diagnosed based on clinical, radiographic, and histopathological findings.

###  Outcome and follow-up

**Figure 5:**
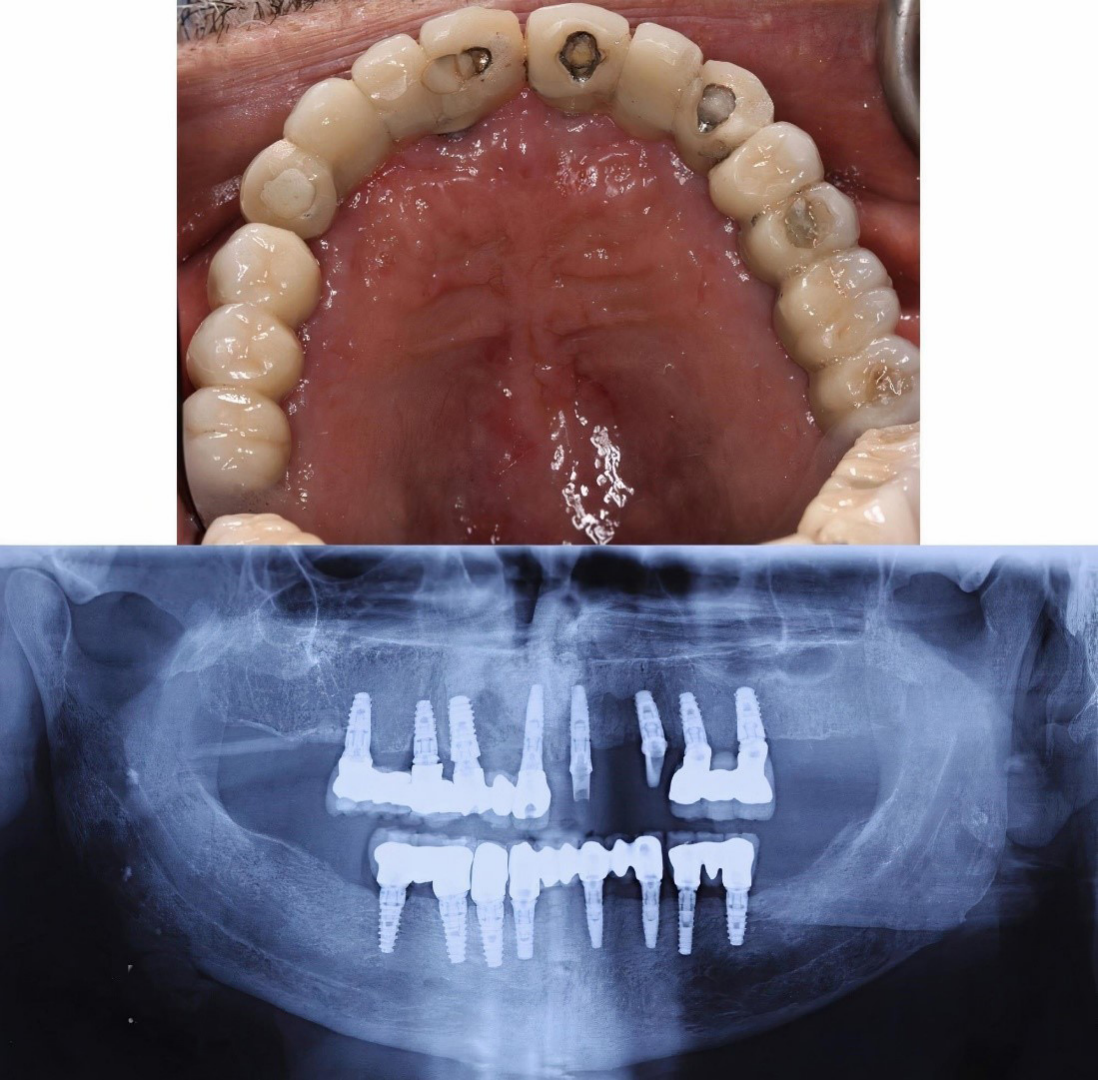
Postoperative clinical and panoramic views of the maxilla after six months

 After ten days, the sutures were removed. The patient reported no pain or discomfort during the healing period and was completely satisfied with the treatment outcome. After six months, a panoramic radiograph was retaken. The radiolucency and swelling had completely disappeared. The prosthesis was removed for a reason other than surgical removal of NPDC (porcelain chipping) (Figure 5).

## Discussion

 Dental implants in the anterior maxilla are of great importance since they restore not only the function but also esthetics.^5^ However, implant placement in this area is not without complications. Although NPDC incidence is not common in the oral cavity, local etiology may induce the proliferation of embryonic tissue remnants.^4,9,10^ Surgical treatment for NPDC depends on the size and location of the cyst. The larger the cyst, the more it is recommended to perform marsupialization first to reduce its size for subsequent enucleation.^8,11^ It has also been suggested to perform autogenous bone grafting in addition to cystectomy.^12^

 In this case report, the cyst was fully removed, and since no problem was observed with the implant, no additional treatment was undertaken. Surgical planning should be based on the findings of CBCT imaging because the measurement of the nasopalatine canal, its location, and the defect morphology are better observed compared to conventional radiographs.

 In this case, either through osteotomy or implant placement, a part of the nasopalatine canal had been disturbed, leading to the proliferation of embryological epithelial remnants of the nasopalatine duct and further cyst formation over time. Casado et al^13^ believe that if the implant is immobile and no signs of periapical lesion are found that may disturb the adjacent teeth or implants, it may be left intact.

 On the other hand, it must be taken into consideration that decontamination of the exposed implants plays a crucial role in re-osseointegration. Because of the rough surface of implants, it is not yet clear whether all the suggested methods, including mechanical and non-mechanical ones, can thoroughly remove the pathogens.^5^

## Conclusion

 In conclusion, some limitations and difficulties may be confronted in the placement of implants in the anterior maxilla. Though reports of nasopalatine duct disturbance are rare, careful case selection and examination, both radiographically and clinically, is highly recommended. In cases of a high chance of interference with the nasopalatine duct, tapered implants, bone grafting, and removal of the neurovascular bundle can be considered.

## Competing Interests

 The authors declare that they have no competing interests.

## Ethical Approval

 Before undertaking treatment, a detailed description of the treatment procedure was provided for the patient. An informed consent was obtained for the treatment and its publication in an article.

## Funding

 None.
